# Whether Patients With Stage Ⅱ/Ⅲ Colorectal Cancer Benefit From Adjuvant Chemotherapy: A Modeling Analysis of Literature Aggregate Data

**DOI:** 10.3389/fphar.2022.826785

**Published:** 2022-02-09

**Authors:** Sijie Zha, Ting Li, Qingshan Zheng, Lujin Li

**Affiliations:** Center for Drug Clinical Research, Shanghai University of Traditional Chinese Medicine, Shanghai, China

**Keywords:** colorectal cancer, adjuvant chemotherapy, survival, modeling, quantitative evaluation, MBMA

## Abstract

**Objective:** This study used model analysis to clarify the benefits and risks of postoperative adjuvant chemotherapy compared with surgery alone in patients with stage II/III colorectal cancer.

**Methods:** Clinical trials involving patients with stage II/III colorectal cancer who underwent surgery alone or those who received post-surgical adjuvant chemotherapy were searched in the PubMed and embase databases. By establishing a survival model, the overall survival (OS) and disease-free survival (DFS) of patients who underwent surgery alone or postoperative adjuvant chemotherapy were quantitatively analyzed to compare the differences between the two. In addition, the incidence of grade 3/4 adverse reactions in the adjuvant chemotherapy group was analyzed using the random effects model in the single-arm meta-analysis.

**Results:** A total of 34 studies containing 33,069 patients were included in the analysis. This study found that postoperative adjuvant chemotherapy can effectively improve the OS and DFS of patients with colorectal cancer. The median OS of the adjuvant chemotherapy group and the surgery-only group was 118.8 months (95% CI: 96.6, 146.6) and 74.6 months (95% CI: 57.8, 96.1) respectively; and median DFS was 86.3 months (95% CI: 67.6, 110.6) and 40.8 months (95% CI: 23.7, 69.6) in the adjuvant chemotherapy and surgery-only groups, respectively. Common grade 3/4 adverse reactions in the adjuvant chemotherapy group include diarrhea, stomatitis, leukopenia, and nausea or vomiting, with an incidence of approximately 3%–6%.

**Conclusion:** Patients with mid-stage colorectal cancer can benefit significantly from postoperative adjuvant chemotherapy. This study provides the necessary quantitative information for decision-making regarding the benefits and risks of receiving adjuvant chemotherapy after resection in patients with colorectal cancer.

## Introduction

Colorectal cancer is one of the most common gastrointestinal malignancies, with the fourth highest incidence of malignancies and the second highest cancer-related mortality worldwide (Center et al., 2009; Salehiniya et al., 2017). Approximately 70%–80% of patients with colorectal cancer are amenable to radical resection; however, postoperative recurrence is the main cause of treatment failure for colorectal cancer, and approximately 19%–28% of patients develop metastases after resection (Jemal et al., 2009; Van Cutsem et al., 2014; Kim et al., 2016; Pinson et al., 2018). The purpose of adjuvant chemotherapy is to eliminate small metastases or micro residual foci that have not been detected during surgery, such that one can improve their prognosis and prolong the survival of patients to obtain more clinical benefits. Adjuvant chemotherapy for colorectal cancer is defined as any 5-FU-based chemotherapy after radical resection of colorectal cancer, including portal vein infusion chemotherapy (Benson et al., 2004).

According to the different depths of invasion, lymph node metastasis, and organ metastasis status of this disease, the current NCCN international guidelines recommend that patients with stage I colorectal cancer should be treated mainly by surgical resection without adjuvant therapy. Patients with stage II colorectal cancer along with other high-risk factors and stage III progressive colorectal cancer should be treated with radical surgery following postoperative adjuvant chemotherapy, while those with advanced or recurrent colorectal cancer are recommended to undergo multidisciplinary evaluation to determine whether there is a chance of resection or radiotherapy, which should be followed by adjuvant chemotherapy (Benson et al., 2020; Benson et al., 2021). However, whether patients with stage II/III disease benefit from adjuvant chemotherapy remains controversial, there is currently no such study for quantitatively evaluating the efficacy of adjuvant chemotherapy regimens after radical surgery for colorectal cancer patients, as recommended in the NCCN guidelines (Carvalho and Glynne-Jones, 2017).

In addition, a series of adverse reactions are also important factors affecting whether a chemotherapy should be performed on patients. For example, the combination of oxaliplatin and fluoropyrimidine will increase the incidence of neutropenia, thrombocytopenia, diarrhea, nausea and depression. There will also be a chronic irreversible peripheral neuropathy (Kuebler et al., 2007; Iveson et al., 2019). Although the survival improves, the incidence of adverse events and the decline in quality of life were also significant for patients. Therefore, quantified comparisons of survival benefit and incidence of adverse events are necessary for physician and patient during a therapy.

Model-based meta-analysis (MBMA) is a quantitative method for evaluating the efficacy or safety of drugs based on traditional meta-analysis using models. Through this method, we can simultaneously correct for multiple influencing factors, deduce the influence of inter-study heterogeneity on the results, and make predictions of drug efficacy at different time points and at different covariate levels (Mandema et al., 2011; Demin et al., 2012). This study aimed to clarify the difference in survival benefit between surgery alone and adjuvant chemotherapy after surgery for patients with stage II/III colorectal cancer using MBMA, as well as evaluate the effect of multiple factors on survival time, aiming to provide reliable quantitative information on the clinical efficacy of adjuvant chemotherapy for colorectal cancer.

## Methods

### Search Strategy

Relevant literature was searched in the PubMed and embase databases, with a search deadline of 29 December 2021, for keywords such as colorectal cancer and adjuvant chemotherapy, with “Or” connecting terms in the same category and “And” connecting terms in different categories. The type of literature included clinical trials, and the language was restricted to English. The specific search strategy is detailed in the Supplements.

### Inclusion and Exclusion Criteria

The inclusion criteria were as follows: 1) the patient had a stage II/III colorectal cancer; 2) the patient had undergone surgical resection; 3) the adjuvant chemotherapy used was the NCCN guideline recommended regimen; and 4) the patient had not received any other treatment prior to enrollment.

The literature exclusion criteria included the following: 1) combination of cancers other than colorectal cancer, 2) patients who received non-adjuvant chemotherapy regimens, and 3) no survival data extracted (Supplementary Figure S1 in Supplements).

### Data Extraction and Quality Evaluation

The following data were extracted using Microsoft Excel (Microsoft Office package, 2019 version): 1) literature characteristics (author, year of publication, clinical trial registration number, etc.); 2) trial characteristics (sample size, dosing method, dosing regimen, etc.); 3) subject characteristics (age, sex, initial status, tumor *in situ*, cancer grade, etc.); and 4) outcome indicators (overall survival [OS], disease-free survival [DFS], and the incidence of grade 3/4 adverse reactions).

The above information was extracted from the data by two investigators independently, with inconsistencies adjudicated by a third investigator. When graphically presenting the data in the literature, the GetData Graph Digitizer software was used to extract the data in the graph. If the error during the extraction between the two researchers was greater than 2%, the data had to be extracted again, and the average value was taken as the final analysis data.

The quality of the literature was evaluated using the Cochrane risk of bias table which includes the evaluation in random sequence generation (Deeks et al., 2019), allocation concealment, performer and participant blinding, outcome assessment blinding, incomplete outcome data, selective publication, and other biases. Among them, we defined other bias as the trial being sponsored by a drug company and the trial being incomparable across subject groups at baseline. Each entry was categorized as low risk, high risk, or unclear risk. The quality of the literature was scored by two researchers independently, and inconsistencies were adjudicated by a third researcher.

### Model Building

Parametric survival models were used to analyze survival data, such as OS and DFS, of patients treated with surgery alone and adjuvant chemotherapy after radical surgery. Visual inspection of the data shows that the survival data was related to the hazard function h(t), which can be interpreted as the instantaneous risk of death at moment t. The hazard function can be described by the equations below.

$$h\left(t\right)=\frac{{\left(\sigma t\sqrt{2\pi }\right)}^{-1}e^{\left(-\frac{1}{2}z^{2}\right)}}{1-\emptyset \left(Z\right)},\;Z=\frac{\ln\left(t\right)-\mu }{\sigma }$$ (1)

h(t) in Eq. 1 conforms to the log-normal distribution, where *μ* and *σ* are the median and standard deviation of the log-normal distribution, respectively (Ding et al., 2020).

Once the base model was constructed, factors that have a potential impact on the model parameters were examined, including subjects’ age, sex, location of carcinoma *in situ*, Dukes’ classification, and the treatment regimen (with or without fluorouracil, with or without fluorouracil combined with calcium folinic acid regimen). Forward inclusion and backward elimination methods were used to screen the covariates using NONMEM software (Mandema et al., 1992; Wahlby et al., 2001). The bound of OFV decreasing in the forward method was set at 3.84 (*p* < 0.05), while in the backward method, the bound was set at 6.63 (*p* < 0.01). The detailed description of the construction of the model is available in Supplements, page five to six.

### Model Evaluation

Several approaches were used to evaluate the model’s performance after the final model was established. First, the goodness-of-fit of the proposed model was evaluated using model diagnostic plots. The model diagnostic plots included scatter plots of observation (OBS) versus population prediction (PRED) and individual predictions (IPRED), conditional weighted residuals (CWRES) versus time and PRED scatter plots, respectively. Second, the visual predictive check (VPC) was used to compare the model predictions with the observed values and evaluate the predictive performance of the model. Finally, the bootstrap method was used to assess the robustness of the model, that is, 1,000 new datasets were taken from the original dataset to obtain the median of the model parameter distribution and its 95% confidence interval (CI), and compared with the estimated values of the model parameters obtained from the original dataset; if they were closer, it indicated that the model was robust and less influenced by individual studies.

### Safety Analysis

The incidence of common grade 3/4 adverse reactions, such as leukopenia, diarrhea, nausea or vomiting, and stomatitis, in the adjuvant chemotherapy group was pooled using a random-effects model in a single-arm meta-analysis to assess the safety of postoperative adjuvant chemotherapy for patients.

### Software

The modeling and simulation processes were performed using NONMEM 7.3 (Level 1.0, ICON Development Solutions, New York, United States), and the model parameters were estimated using first-order conditional estimation. Meta-analysis and graphical visualization were performed using R software (version 4.0.3, The R Foundation of Statistical Computing, Vienna, Austria). The literature quality assessment was performed using RevMan (version 5.4, Nordic Cochrane Center, Copenhagen, Denmark).

## Results

### Characteristics of the Included Studies

The study ultimately included 34 publications that enrolled 33,069 patients, which consisted of 21 publications (23 treatment arms, sample size of 7,020) in the surgery-only group and 31 publications (48 treatment arms, sample size of 26,049) in the postoperative adjuvant chemotherapy group (Table 1).

**TABLE 1:** Brief characteristics of included studies.

	Control	ACT	Overall
Number of trials (arms)	21 (23)	31 (48)	34 (71)
Total sample size	7,020	26,049	33,069
Age, yr, median (min-max)	62 (15–86)	62 (15–95)	62 (15–95)
Male, %, median (min-max)	53.5 (14.2–70.5)	56.0 (42.7–73.3)	56.0 (14.2–73.3)
Primary tumor, %, median (min-max)			
Colon	55.5 (12.7–71.0)	67.8 (30.6–100)	61.1 (12.7–100)
Rectum	39.0 (11.7–71.0)	32.2 (0–65.8)	36.7 (0–65.8)
Dukes’ stage, %, median (min-max)			
Dukes’ B	43 (0–92)	41 (0–91)	41 (0–92)
Dukes’ C	38 (8–100)	47 (8–100)	42 (8–100)

ACT, indicates adjuvant chemotherapy; Control, indicates surgery alone.

From the publications, we extracted data on OS, the gold standard for assessing clinical benefit in oncology, and DFS, the most common endpoint for evaluating adjuvant therapy after radical surgery. A total of 32 publications reported data on OS at different time points, comprising 67 trial arms (23 in the surgery-only group and 44 in the adjuvant chemotherapy group); 20 publications reported data on DFS, comprising 42 trial arms (11 in the surgery-only group and 31 in the adjuvant chemotherapy group).

The mean age of patients in the 34 studies was 54–68 years (median age, 62 years), with a median male prevalence of 56.0% (14.2%–73.3%). The proportion of primary tumors located in the colon was 12.7%–100% (median, 61.1%), and the proportion of primary tumors located in the rectum was 0%–65.8% (median, 36.7%). Among the included studies, 9 (26.5%) were of high quality, 25 (73.5%) were of medium quality, and 0 were of low quality (Supplementary Table S1 & Supplementary Figure S2 in Supplements).

### Model Building and Evaluation

The results showed that the log-normal model had lower OFV values and a smaller relative standard error (RSE) % of the model parameters. Therefore, the log-normal model was selected to fit the OS and DFS data. In covariate screening, we did not find any factors that had a significant impact on the parameters in the OS and DFS models. The estimated values of the final model parameters are listed in Table 2.

**TABLE 2:** Parameter estimations of model.

	Overall survival model				Disease-free survival model			
	Final model		Bootstrap (993/1,000)		Final model		Bootstrap (996/1,000)	
Parameters	Value	RSE%	Median	95%CI	Value	RSE%	Median	95%CI
SIGM1 (ACT)	1.44	3.8	1.44	1.32–1.55	2.08	2.4	2.08	1.98–2.17
SIGM2(CONTROL)	1.34	5.9	1.35	1.20–1.53	1.90	8.1	1.89	1.52–2.26
MU1(ACT)	4.87	2.1	4.86	4.69–5.08	4.90	2.5	4.90	4.65–5.13
MU2(CONTROL)	4.35	2.8	4.36	4.12–4.62	4.05	6.4	4.04	3.49–4.67
Variability parameters								
η(SIGM),%	23.6	13.9	22.9	16.9–44.2	13.2	32.8	12.4	3.99–27.7
η(MU),%	12.9	8.9	12.7	10.4–15.2	15.3	11.7	14.7	11.1–18.8
ε	1.03	9.8	1.03	0.85–1.23	1.63	9.6	1.62	1.31–1.93

ACT, indicates adjuvant chemotherapy; CONTROL, indicates surgery alone; *η* is the inter-study variability of pharmacodynamic parameter; ε is the residual error. CI, confidence interval; RSE, relative standard error.

The RSE of the model parameters in both the OS and DFS models was small, indicating that the model parameter estimates were relatively stable. The bootstrap method with 1,000 iterations converged successfully 993 times and 996 times, respectively, with the 95% CIs very close to the parameter estimates of the final OS and DFS models, suggesting that the final models were robust and influenced by the data of individual studies was relatively small.

**FIGURE 1:**
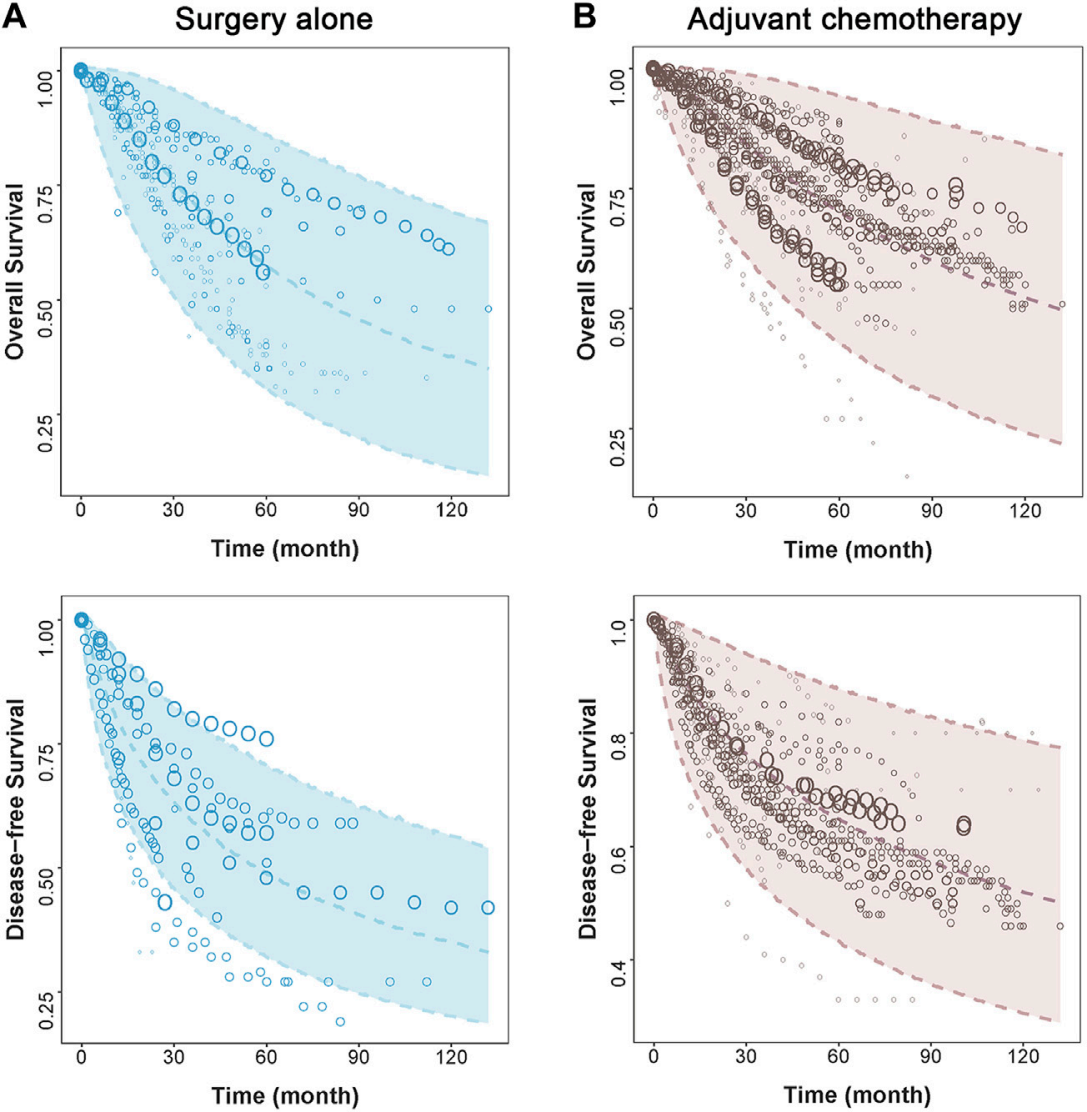
Visual predictive check of the final model. The points represent observed survival data, and symbol size is proportional to sample size. The shade area is the model predicted 95% CI of the curve. The dashed lines are the model-predicted 2.5th, 50th, and 97.5th percentiles of survival. The two figures on the left side represent **(A)** surgery alone group, and two figures on the right side represent **(B)** adjuvant chemotherapy group.

The model diagnostic plots (Supplementary Figure S3, S4 in Supplements) showed that the model-predicted values for OS and DFS fit well with the observed data without significant bias. The visual predictive check (VPC) plots showed that the 95% CIs predicted by the OS and DFS models included most of the measured values, suggesting that the models have good predictive ability (Figure 1).

### Model Simulation

Based on the final model, the typical OS and DFS values and their 95% CIs were simulated for the surgery-only and adjuvant chemotherapy groups, showing a median OS of 118.8 months (95% CI: 96.6, 146.6) and 74.6 months (95% CI: 57.8, 96.1) for the adjuvant chemotherapy and surgery-only groups, respectively, with the former being 1.6 times higher than the latter; 5-years survival rates of 71.6% (95% CI: 65.1, 77.7) and 57.8% (95% CI: 48.7, 67.2), respectively; and 10-years survival rates of 49.7% (95% CI: 42.9, 56.8) and 33.1% (95% CI: 25.6, 41.8), respectively.

**FIGURE 2:**
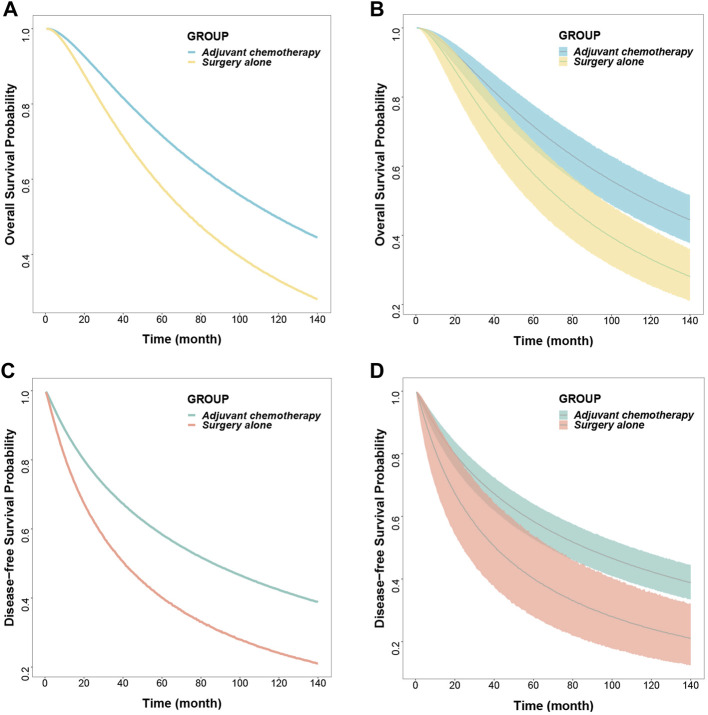
Predicted typical time course **(A) (C)** and 95% confidence interval **(B) (D)** of overall survival and disease-free survival.

The median DFS rates were 86.3 months (95% CI: 67.6, 110.6) and 40.8 months (95% CI: 23.7, 69.6) months in the adjuvant chemotherapy group and the surgery-only group, respectively; 5-years survival rates of 58.5% (95% CI: 52.9, 63.9) and 40.1% (95% CI: 28.1, 53.8), respectively; and 10-years survival rates of 42.3% (95% CI: 36.7, 48.0) and 24.1% (95% CI: 14.8, 35.7), respectively (Figure 2; Table 3).

**TABLE 3:** The predicted typical time course with 95% confidence interval of OS and DFS model.

	Median Overall Survival (month)	Five-year Overall Survival (%)	Ten-year Overall Survival (%)
Surgery	74.6 (57.8, 96.1)	57.8 (48.7, 67.2)	33.1 (25.6, 41.8)
Surgery + ACT	118.8 (96.6, 146.6)	71.6 (65.1, 77.7)	49.7 (42.9, 56.8)
	**Median Disease-free Survival (month)**	**Five-year Disease-free Survival (%)**	**Ten-year Disease-free Survival (%)**
Surgery	40.8 (23.7, 69.6)	40.1 (28.1, 53.8)	24.1 (14.8, 35.7)
Surgery + ACT	86.3 (67.6, 110.6)	58.5 (52.9, 63.9)	42.3 (36.7, 48.0)

ACT, indicates adjuvant chemotherapy.

### Safety Analysis

A total of 18 publications reported the incidence of grade 3/4 adverse reactions, and the results showed that the incidence of grade 3/4 diarrhea, leukopenia, stomatitis, and nausea or vomiting in the postoperative adjuvant chemotherapy group was 6% (95% CI: 4, 9), 3% (95% CI: 1, 6), 4% (95% CI: 3, 8), and 4% (95% CI: 3, 6), respectively (Supplementary Figure S5 in Supplements).

## Discussion

In recent years, despite a variety of emerging therapies for patients with colorectal cancer, such as radiation therapy, targeted therapy, and preoperative neoadjuvant therapy, adjuvant chemotherapy remains the basic therapy for this disease because of its wide application in various clinical situations. Disputes still exists on whether patients with middle-stage colorectal cancer can benefit from postoperative adjuvant chemotherapy, while what has already been agreed upon is that those with early-stage colorectal cancer are not advised with an adjuvant chemotherapy, and those with advanced ones should be treated with palliative care (Carvalho and Glynne-Jones, 2017; Dekker et al., 2019; Benson et al., 2020; Benson et al., 2021). Therefore, it is essential to conduct a quantitative comparison between adjuvant chemotherapy and surgical treatment alone in patients with middle-stage colorectal cancer.

Previous studies have focused on whether patients with other specific types of colorectal cancer benefit from adjuvant chemotherapy, for example, whether those with lung metastases benefit from adjuvant chemotherapy, whether those with peritoneal metastases benefit from intraperitoneal hyperthermia chemotherapy, and whether there is a difference between long-term prognosis from three drugs and two drugs in palliative first-line treatment (Gill et al., 2004; Group et al., 2007; E. and; Mitry, 2008; Zhang et al., 2019)_ENREF_20. However, few studies have been done to quantitatively describe the survival benefits for middle-stage colorectal cancer patients from a adjuvant chemotherapy. A Meta analysis (Böckelman et al., 2015) found that only patients with stage Ⅲ colorectal cancer could benefit from adjuvant chemotherapy, while the 5-years DFS of patients with stage II colorectal cancer with adjuvant chemotherapy was even lower than that without adjuvant chemotherapy. However, this study only included the literature published in 2005–2013 for analysis, and did not analyze OS, the conclusions of the study may be biased.

In this study, we established a survival model with a hazard function to reflect the difference among middle-stage colorectal cancer patients being treated with drugs recommended by the NCCN guideline and explored whether they benefit from adjuvant chemotherapy. We found a significant difference in OS and DFS between patients who received surgical treatment only and those who received adjuvant chemotherapy after surgery. We also found that the difference in DFS was more significant than that in OS, in which the adjuvant chemotherapy group had a 2.1 times larger DFS than those in the surgery group. Compared with previous studies, this study not only confirmed that patients with middle-stage colorectal cancer can benefit from adjuvant chemotherapy, but also can predict the survival time at any arbitrary time point, not limited to the median survival time and 1-year survival rate, so as to show the benefits of adjuvant chemotherapy in the whole time period.

There is a clinical debate on whether it deserves to use the adjuvant chemotherapy on patients compared to its safety issues (Yothers et al., 2011; Carvalho and Glynne-Jones, 2017; Zhang et al., 2019; La Regina et al., 2020). Although one may live a little longer after adjuvant chemotherapy, it depends on various adverse events, and whether one can live with a higher quality life matters more. In this study, we could not find any record of adverse events in the surgery-only group; thus, a single-arm meta-analysis was applied to the data of adverse events in the adjuvant chemotherapy group. The results showed that the incidence of regular grade 3/4 adverse events was no more than 6% in the adjuvant chemotherapy group, which included diarrhea and stomatitis (4%), leukopenia (3%), and nausea or vomiting (4%). The results can be useful for decision makers in determining whether adjuvant chemotherapy should be administered after surgery.

Reports have indicated that factors such as age and the location of carcinoma *in situ* can affect the survival of patients with colorectal cancer (Holch et al., 2017; Nikolic et al., 2021). Researchers have also reported that those under the age of 65 years can survive better than those older than 65 years. Moreover, some reported that patients with rectal cancer had a higher OS than those who experienced colon cancer in a 5-years range (Gill et al., 2004; Schmoll et al., 2014). However, this study did not find any covariate that had a significant impact on survival rate, including age, sex, initial status, tumor *in situ*, and cancer grade. The reason may be that our research is based on literature aggregate data, which to some extent masks individual differences and reduces the chances of finding covariates. Besides that, because the missing rate of factors is more than 30%, such as MSI status, perineural invasion, histologic grade, and serum CEA level, the covariates can not be investigated. This is one of the limitations of this study. Beside that, due to multifarious medication regimens and chemotherapy cycles, it is hard for us to make a more detailed category for our enrolled literatures. Finally, only studies published in English were included, therefore, the risk of publication bias could be present.

## Conclusion

This study quantified the survival benefit of adjuvant chemotherapy for colorectal cancer and found that postoperative adjuvant chemotherapy significantly prolonged patients’ OS and DFS compared with surgery alone, providing quantitative evidence that patients with intermediate colorectal cancer benefit from adjuvant chemotherapy.

## Data Availability

The original contributions presented in the study are included in the article/Supplementary Material, further inquiries can be directed to the corresponding authors.

## References

[B1] Benson A. B. 3rd Schrag D. Somerfield M. R. Cohen A. M. Figueredo A. T. Flynn P. J. (2004). American Society of Clinical Oncology Recommendations on Adjuvant Chemotherapy for Stage II colon Cancer. J. Clin. Oncol. 22 (16), 3408–3419. 10.1200/JCO.2004.05.063 15199089

[B2] Benson A. B. Venook A. P. Al-Hawary M. M. Arain M. A. Chen Y. J. Ciombor K. K. (2020). NCCN Guidelines Insights: Rectal Cancer, Version 6.2020. J. Natl. Compr. Canc Netw. 18 (7), 806–815. 10.6004/jnccn.2020.0032 32634771

[B3] Benson A. B. Venook A. P. Al-Hawary M. M. Arain M. A. Chen Y.-J. Ciombor K. K. (2021). Colon Cancer, Version 2.2021, NCCN Clinical Practice Guidelines in Oncology. J. Natl. Compr. Canc Netw. 19 (3), 329–359. 10.6004/jnccn.2021.0012 33724754

[B4] Böckelman C. Engelmann B. E. Kaprio T. Hansen T. F. Glimelius B. (2015). Risk of Recurrence in Patients with colon Cancer Stage II and III: a Systematic Review and Meta-Analysis of Recent Literature. Acta Oncol. 54 (1), 5–16. 10.3109/0284186x.2014.975839 25430983

[B5] Carvalho C. Glynne-Jones R. (2017). Challenges behind Proving Efficacy of Adjuvant Chemotherapy after Preoperative Chemoradiation for Rectal Cancer. Lancet Oncol. 18 (6), e354–e363. 10.1016/s1470-2045(17)30346-7 28593861

[B6] Center M. M. Jemal A. Smith R. A. Ward E. (2009). Worldwide Variations in Colorectal Cancer. CA Cancer J. Clin. 59 (6), 366–378. 10.3322/caac.20038 19897840

[B7] Deeks J. J. Higgins J. P. Altman D. G. (2019). Analysing Data and Undertaking Meta-Analyses. Cochrane Handbook Syst. Rev. Interventions 6, 241–284. 10.1002/9781119536604.ch10

[B8] Dekker E. Tanis P. J. Vleugels J. L. A. Kasi P. M. Wallace M. B. (2019). Colorectal Cancer. Lancet 394 (10207), 1467–1480. 10.1016/s0140-6736(19)32319-0 31631858

[B9] Demin I. Hamrén B. Luttringer O. Pillai G. Jung T. (2012). Longitudinal Model-Based Meta-Analysis in Rheumatoid Arthritis: an Application toward Model-Based Drug Development. Clin. Pharmacol. Ther. 92 (3), 352–359. 10.1038/clpt.2012.69 22760002

[B10] Ding J. Thuy Thuong Thuong N. Pham T. V. Heemskerk D. Pouplin T. Tran C. T. H. (2020). Pharmacokinetics and Pharmacodynamics of Intensive Antituberculosis Treatment of Tuberculous Meningitis. Clin. Pharmacol. Ther. 107 (4), 1023–1033. 10.1002/cpt.1783 31956998 PMC7158205

[B11] Gandomani H. S. Yousefi S. M. Aghajani M. Mohammadian-Hafshejani A. Tarazoj A. A. Pouyesh V. (2017). Colorectal Cancer in the World: Incidence, Mortality and Risk Factors. Biomed. Res. Ther. 4 (10), 1656. 10.15419/bmrat.v4i10.372

[B12] Gill S. Loprinzi C. L. Sargent D. J. Thomé S. D. Alberts S. R. Haller D. G. (2004). Pooled Analysis of Fluorouracil-Based Adjuvant Therapy for Stage II and III colon Cancer: Who Benefits and by How Much?. J. Clin. Oncol. 22 (10), 1797–1806. 10.1200/JCO.2004.09.059 15067028

[B13] Group Q. C. Gray R. Barnwell J. McConkey C. Hills R. K. Williams N. S. (2007). Adjuvant Chemotherapy versus Observation in Patients with Colorectal Cancer: a Randomised Study. Lancet 370 (9604), 2020–2029. 10.1016/s0140-6736(07)61866-2 18083404

[B14] Holch J. W. Ricard I. Stintzing S. Modest D. P. Heinemann V. (2017). The Relevance of Primary Tumour Location in Patients with Metastatic Colorectal Cancer: A Meta-Analysis of First-Line Clinical Trials. Eur. J. Cancer 70, 87–98. 10.1016/j.ejca.2016.10.007 27907852

[B15] Iveson T. Boyd K. A. Kerr R. S. Robles-Zurita J. Saunders M. P. Briggs A. H. (2019). 3-month versus 6-month Adjuvant Chemotherapy for Patients with High-Risk Stage II and III Colorectal Cancer: 3-year Follow-Up of the SCOT Non-inferiority RCT. Health Technol. Assess. 23 (64), 1–88. 10.3310/hta23640 PMC6936167 31852579

[B16] Jemal A. Siegel R. Ward E. Hao Y. Xu J. Thun M. J. (2009). Cancer Statistics, 2009. CA Cancer J. Clin. 59, 225–249. 10.3322/caac.20006 19474385

[B17] Kim C. W. Baek J. H. Choi G. S. Yu C. S. Kang S. B. Park W. C. (2016). The Role of Primary Tumor Resection in Colorectal Cancer Patients with Asymptomatic, Synchronous Unresectable Metastasis: Study Protocol for a Randomized Controlled Trial. Trials 17, 34. 10.1186/s13063-016-1164-0 26782254 PMC4717596

[B18] Kuebler J. P. Wieand H. S. O'Connell M. J. Smith R. E. Colangelo L. H. Yothers G. (2007). Oxaliplatin Combined with Weekly Bolus Fluorouracil and Leucovorin as Surgical Adjuvant Chemotherapy for Stage II and III colon Cancer: Results from NSABP C-07. J. Clin. Oncol. 25 (16), 2198–2204. 10.1200/jco.2006.08.2974 17470851

[B19] La Regina D. Mongelli F. Fasoli A. Lollo G. Ceppi M. Saporito A. (2020). Clinical Adverse Events after Endoscopic Resection for Colorectal Lesions: A Meta-Analysis on the Antibiotic Prophylaxis. Dig. Dis. 38 (1), 15–22. 10.1159/000502055 31408875

[B20] Mandema J. W. Gibbs M. Boyd R. A. Wada D. R. Pfister M. (2011). Model-based Meta-Analysis for Comparative Efficacy and Safety: Application in Drug Development and beyond. Clin. Pharmacol. Ther. 90 (6), 766–769. 10.1038/clpt.2011.242 22089340

[B21] Mandema J. W. Verotta D. Sheiner L. B. (1992). Building Population Pharmacokinetic-Ppharmacodynamic Models. I. Models for Covariate Effects. J. Pharmacokinet. Biopharm. 20, 511–528. 10.1007/BF01061469 1287200

[B22] Mitry E. (2008). Levamisole and Fluorouracil for Adjuvant Therapy of Resected colon Carcinoma. Côlon & Rectum 2 (1), 37–39. 10.1056/NEJM199002083220602

[B23] Nikolic N. Radosavljevic D. Gavrilovic D. Nikolic V. Stanic N. Spasic J. (2021). Prognostic Factors for Post-Recurrence Survival in Stage II and III Colorectal Carcinoma Patients. Medicina (Kaunas) 57 (10), 1108. 10.3390/medicina57101108 34684145 PMC8538010

[B24] Pinson H. Cosyns S. Ceelen W. P. (2018). The Impact of Surgical Resection of the Primary Tumor on the Development of Synchronous Colorectal Liver Metastasis: a Systematic Review. Acta Chir Belg. 118 (4), 203–211. 10.1080/00015458.2018.1446602 29783886

[B25] Schmoll H. J. Twelves C. Sun W. O'Connell M. J. Cartwright T. McKenna E. (2014). Effect of Adjuvant Capecitabine or Fluorouracil, with or without Oxaliplatin, on Survival Outcomes in Stage III colon Cancer and the Effect of Oxaliplatin on post-relapse Survival: a Pooled Analysis of Individual Patient Data from Four Randomised Controlled Trials. Lancet Oncol. 15 (13), 1481–1492. 10.1016/s1470-2045(14)70486-3 25456367 PMC4435787

[B26] Van Cutsem E. Cervantes A. Nordlinger B. Arnold D. Group E. G. W. (2014). Metastatic Colorectal Cancer: ESMO Clinical Practice Guidelines for Diagnosis, Treatment and Follow-Up. Ann. Oncol. 25 (3), 1–9. 10.1093/annonc/mdu260 25190710

[B27] Wählby U. Jonsson E. N. Karlsson M. O. (2001). Assessment of Actual Significance Levels for Covariate Effects in NONMEM. J. Pharmacokinet. Pharmacodynamics 28 (3), 231–252. 10.1023/a:1011527125570 11468939

[B28] Yothers G. O'Connell M. J. Allegra C. J. Kuebler J. P. Colangelo L. H. Petrelli N. J. (2011). Oxaliplatin as Adjuvant Therapy for colon Cancer: Updated Results of NSABP C-07 Trial, Including Survival and Subset Analyses. J. Clin. Oncol. 29 (28), 3768–3774. 10.1200/JCO.2011.36.4539 21859995 PMC3188282

[B29] Zhang C. Tan Y. Xu H. (2019). Does Adjuvant Chemotherapy Improve the Prognosis of Patients after Resection of Pulmonary Metastasis from Colorectal Cancer? A Systematic Review and Meta-Analysis. Int. J. Colorectal Dis. 34 (10), 1661–1671. 10.1007/s00384-019-03362-7 31446479

