# Supplementary material for: Whether Patients With Stage Ⅱ/Ⅲ Colorectal Cancer Benefit From Adjuvant Chemotherapy: A Modeling Analysis of Literature Aggregate Data
Source: Front Pharmacol. 2022 Feb 9;13:826785. doi: 10.3389/fphar.2022.826785 (PMC8864068; doi:10.3389/fphar.2022.826785)

**Supplementary**

**Search strategy**

**PubMed**

| No. | Query | Results | Date |
| --- | --- | --- | --- |
| #1 | ((((CRC[Title/Abstract]) OR colorectal cancer[Title/Abstract]) OR (carcinoma of colon[Title/Abstract] AND rectum[Title/Abstract])) OR (cancer of colon[Title/Abstract] AND rectum[Title/Abstract])) | 119855 | 29-Dec-21 |
| #2 | chemotherapy[Title/Abstract] | 403498 | 29-Dec-21 |
| #3 | #1 AND #2 | 16304 | 29-Dec-21 |
| #4 | Filters:Clinical Trial | 2198 | 29-Dec-21 |

**Embase**

| #1 | 'colorectal cancer':ab,ti OR 'carcinoma of colon':ab,ti OR 'carcinoma of rectum':ab,ti OR 'cancer of colon':ab,ti OR 'cancer of rectum':ab,ti | 168581 | 29-Dec-21 |
| --- | --- | --- | --- |
| #2 | chemotherapy:ab,ti | 640814 | 29-Dec-21 |
| #3 | #1 AND #2 | 26246 | 29-Dec-21 |
| #4 | #3 AND 'clinical trial'/de | 3366 | 29-Dec-21 |

**List of included studies**

1. Center MM, Jemal A, Smith RA, Ward E. Worldwide variations in colorectal cancer. CA Cancer J Clin. 2009;59(6):366-378.

2. Salehiniya H, Pouyesh V, Tarazoj AA, et al. Colorectal cancer in the world: incidence, mortality and risk factors. Biomedical Research and Therapy. 2017;4(10).

3. Jemal A, R S, Xu J, Ward E. Cancer Statistics. CA Cancer J Clin. 2009;59:1-24.

4. Pinson H, Cosyns S, Ceelen WP. The impact of surgical resection of the primary tumor on the development of synchronous colorectal liver metastasis: a systematic review. Acta Chir Belg. 2018;118(4):203-211.

5. Van Cutsem E, Cervantes A, Nordlinger B, Arnold D, Group EGW. Metastatic colorectal cancer: ESMO Clinical Practice Guidelines for diagnosis, treatment and follow-up. Ann Oncol. 2014;25 Suppl 3:iii1-9.

6. Kim CW, Baek JH, Choi GS, et al. The role of primary tumor resection in colorectal cancer patients with asymptomatic, synchronous unresectable metastasis: Study protocol for a randomized controlled trial. Trials. 2016;17:34.

7. Benson AB, 3rd, Schrag D, Somerfield MR, et al. American Society of Clinical Oncology recommendations on adjuvant chemotherapy for stage II colon cancer. J Clin Oncol. 2004;22(16):3408-3419.

8. Benson AB, Venook AP, Al-Hawary MM, et al. Colon Cancer, Version 2.2021, NCCN Clinical Practice Guidelines in Oncology. J Natl Compr Canc Netw. 2021;19(3):329-359.

9. Benson AB, Venook AP, Al-Hawary MM, et al. NCCN Guidelines Insights: Rectal Cancer, Version 6.2020. J Natl Compr Canc Netw. 2020;18(7):806-815.

10. Carvalho C, Glynne-Jones R. Challenges behind proving efficacy of adjuvant chemotherapy after preoperative chemoradiation for rectal cancer. The Lancet Oncology. 2017;18(6):e354-e363.

11. Iveson T, Boyd KA, Kerr RS, et al. 3-month versus 6-month adjuvant chemotherapy for patients with high-risk stage II and III colorectal cancer: 3-year follow-up of the SCOT non-inferiority RCT. Health Technol Assess. 2019;23(64):1-88.

12. Oxaliplatin Combined With Weekly Bolus Fluorouracil and Leucovorin As Surgical Adjuvant Chemotherapy for Stage II and III Colon Cancer: Results From NSABP C-07. Journal of Clinical Oncology. 2007;25(16):2198-2204.

13. Mandema JW, Gibbs M, Boyd RA, Wada DR, Pfister M. Model-based meta-analysis for comparative efficacy and safety: application in drug development and beyond. Clin Pharmacol Ther. 2011;90(6):766-769.

14. Demin I, Hamren B, Luttringer O, Pillai G, Jung T. Longitudinal model-based meta-analysis in rheumatoid arthritis: an application toward model-based drug development. Clin Pharmacol Ther. 2012;92(3):352-359.

15. Deeks JJ, Higgins JPT, Altman DG. Analysing data and undertaking meta-analyses. In:2019:241-284.

16. Ding J, Thuy Thuong Thuong N, Pham TV, et al. Pharmacokinetics and Pharmacodynamics of Intensive Antituberculosis Treatment of Tuberculous Meningitis. Clin Pharmacol Ther. 2020;107(4):1023-1033.

17. Mandema JW, Verotta D, Sheiner LB. Building population pharmacokineticpharmacodynamic models. I. Models for covariate effects. Journal of Pharmacokinetics & Biopharmaceutics. 1992;20.

18. Ulrika, Wählby, E., et al. Assessment of Actual Significance Levels for Covariate Effects in NONMEM. Journal of Pharmacokinetics & Pharmacodynamics. 2001.

19. Dekker E, Tanis PJ, Vleugels JLA, Kasi PM, Wallace MB. Colorectal cancer. The Lancet. 2019;394(10207):1467-1480.

20. Zhang C, Tan Y, Xu H. Does adjuvant chemotherapy improve the prognosis of patients after resection of pulmonary metastasis from colorectal cancer? A systematic review and meta-analysis. Int J Colorectal Dis. 2019;34(10):1661-1671.

21. E., Mitry. Levamisole and fluorouracil for adjuvant therapy of resected colon carcinoma. Côlon & Rectum. 2008;2(1):37-39.

22. Gill S, Loprinzi CL, Sargent DJ, et al. Pooled analysis of fluorouracil-based adjuvant therapy for stage II and III colon cancer: who benefits and by how much? J Clin Oncol. 2004;22(10):1797-1806.

23. Group QC. Adjuvant chemotherapy versus observation in patients with colorectal cancer: a randomised study. The Lancet. 2007;370(9604):2020-2029.

24. Böckelman C, Engelmann BE, Kaprio T, Hansen TF, Glimelius B. Risk of recurrence in patients with colon cancer stage II and III: a systematic review and meta-analysis of recent literature. Acta Oncol. 2015;54(1):5-16.

25. Yothers G, O'Connell MJ, Allegra CJ, et al. Oxaliplatin as adjuvant therapy for colon cancer: updated results of NSABP C-07 trial, including survival and subset analyses. J Clin Oncol. 2011;29(28):3768-3774.

26. La Regina D, Mongelli F, Fasoli A, et al. Clinical Adverse Events after Endoscopic Resection for Colorectal Lesions: A Meta-Analysis on the Antibiotic Prophylaxis. Dig Dis. 2020;38(1):15-22.

27. Nikolic N, Radosavljevic D, Gavrilovic D, et al. Prognostic Factors for Post-Recurrence Survival in Stage II and III Colorectal Carcinoma Patients. Medicina (Kaunas). 2021;57(10).

28. Holch JW, Ricard I, Stintzing S, Modest DP, Heinemann V. The relevance of primary tumour location in patients with metastatic colorectal cancer: A meta-analysis of first-line clinical trials. Eur J Cancer. 2017;70:87-98.

29. Schmoll H-J, Twelves C, Sun W, et al. Effect of adjuvant capecitabine or fluorouracil, with or without oxaliplatin, on survival outcomes in stage III colon cancer and the effect of oxaliplatin on post-relapse survival: a pooled analysis of individual patient data from four randomised controlled trials. The Lancet Oncology. 2014;15(13):1481-1492.

**
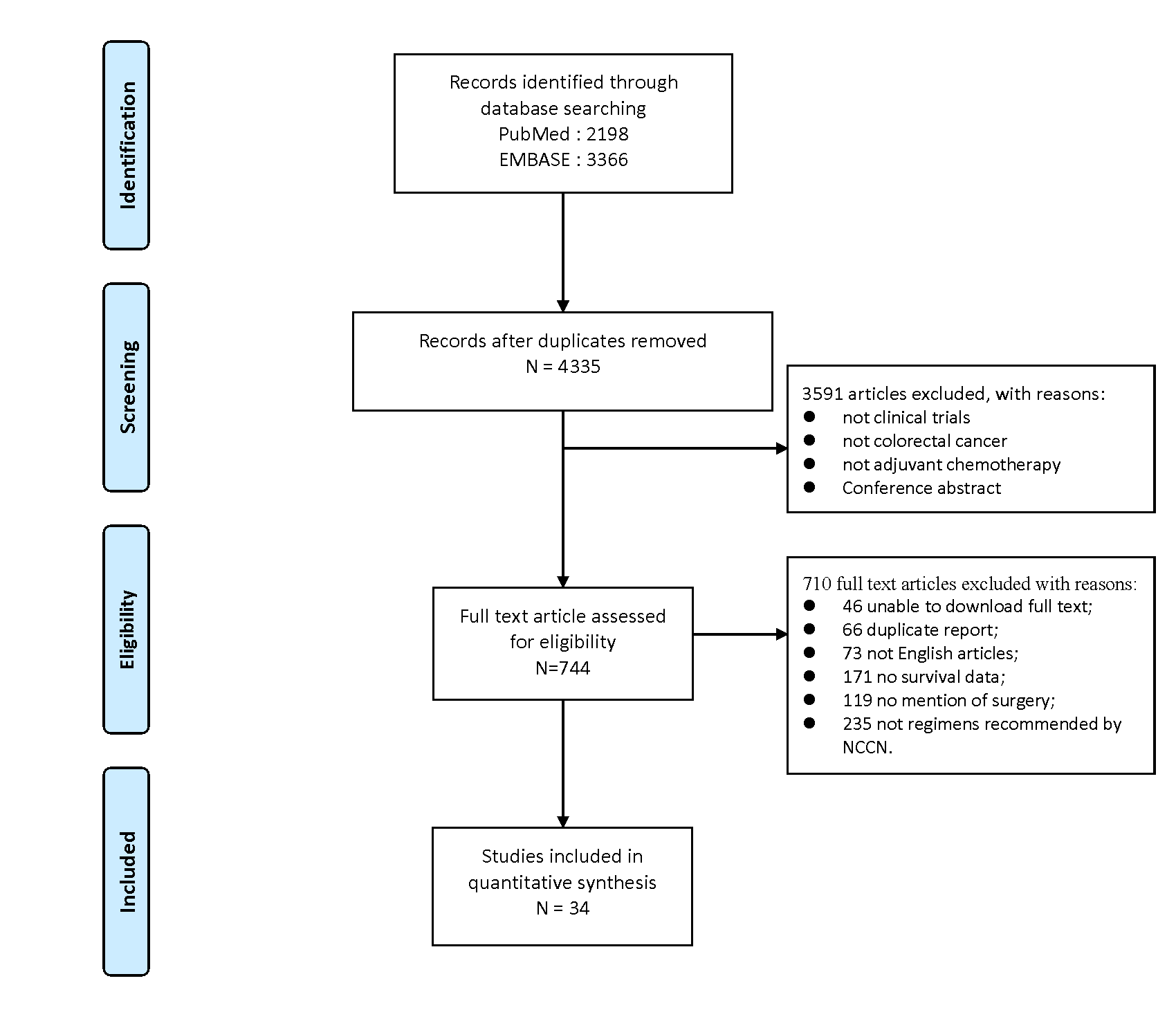
**

**Figure S1.** Flow chart demonstrating the inclusion and exclusion of studies into the analysis. NCCN indicates National Comprehensive Cancer Network.

**Model building**

Parametric survival models were used to analyze survival data, such as OS and DFS, of patients treated with surgery alone and adjuvant chemotherapy after radical surgery. The survival model was related to the hazard function h(t), which can be interpreted as the instantaneous risk of death at moment t. The relationship between the survival model and the hazard function was as follows:

*Survival model* $S(t)=exp(-\int_{0}^{t} h(t)dt)$ (1)

This study evaluated four different hazard functions as follows:

*Constant* $h\left( t \right)=\lambda$ (2)

*Gompertz* $h\left( t \right)=\lambda\cdot exp(\beta\cdot t)$ (3)

*Weibull* $h\left( t \right)=\lambda\cdot exp(\beta\cdot\ln\left( t \right))$ (4)

*Lognormal* $h\left( t \right)=\frac{\left( \sigma t\sqrt{2\pi} \right)^{-1}e^{\left( -\frac{1}{2}z^{2} \right)}}{1-\emptyset\left( Z \right)}$*,* (5)

$Z=\frac{\ln\left( t \right)-\mu}{\sigma}$

In Equations 2–5, h(t) represent the value at risk at time t, and *λ* and *β* denote the risk at moment 0 and the coefficient of risk over time, respectively. h(t) in Equation 5 conforms to the log-normal distribution, where *μ* and *σ* are the median and standard deviation of the log-normal distribution, respectively. The best hazard model was selected based on the minimum value of the objective function, standard error of the model parameter estimation, and goodness-of-fit of the model.

When conditions permit, the inter-trial variation (Equation 6) and residuals (Equation 7) were added to the model parameters to account for differences between observed and model-predicted values. The inter-study variability (η) and residual variability (ε) could be explained by a random effects model (Equation 6–7), where inter-study variability is introduced into the model parameters in an exponential form and residual variability is chosen as an additive model:

$P_{i}=P_{pop}\times e^{\eta_{i}}$ (6)

${Obs}_{j,i}={Pred}_{j,i}+\mathrm{SE}_{j,i}\times\varepsilon_{j,i}$ (7)

${SE}_{j,i}=\sqrt{\frac{{Obs}_{j,i}\times(1-{Obs}_{j,i})}{N_{j,i}}}$ (8)

In Equation 6, $P_{i}$ is the individual value of the model parameter for study i, and $P_{\mathrm{pop}}$ is the typical population value of the model parameter. $\eta_{i}$ is the within-trial variation of the model parameter, which conforms to a normal distribution with mean 0 and variance ω_i_^2^. In Equation 7, $\mathrm{Obs}_{j,i}$ is the measured value of survival at time j in study i. $\mathrm{Pred}_{j,i}$ is the predicted value of survival at time j of study i. $\varepsilon_{j,i}$ is the residual of time j of study i, which conforms to a normal distribution with mean 0 and variance σ^2^. $\varepsilon_{j,i}$ is corrected by the standard error of survival at time j of study i (SE_j,i_), which is considered to be smaller with the decrease in the standard error. The formula for SE_i,j_ is given in Equation 8, where N_j,i_ is the sample size of time j in study i.

Once the base model was constructed, factors that have a potential impact on the model parameters were examined, including subjects’ age, sex, location of carcinoma *in situ*, Dukes' classification, and the treatment regimen (with or without fluorouracil, with or without fluorouracil combined with calcium folinic acid regimen). When a variable was missing and the proportion of missing values was less than 30%, the median of the remaining studies for that variable was used to replace the missing data. Covariate analysis was not used when the proportion of missing values was greater than 30%. All covariates were introduced into the model in an exponential form, which was multiplied by the hazard function h(t). Forward and backward methods were used to screen the covariates to confirm the covariates that eventually entered the model. The bound of OFV decreasing in the forward method was set at 3.84 (P<0.05), while in the backward method, the bound was set at 6.63 (P<0.01).

**Table S1.** Summary of included studies. NA: not mentioned.

| **Study** | **Treatment** | **Age** | **Male** | **Sample** | **Stage** | **Chemotherapy regimens** | **Surgery** | **Random** | **Blind** |
| --- | --- | --- | --- | --- | --- | --- | --- | --- | --- |
|  |  | **(year, median)** | **(%)** | **size** |  |  | **(Yes/No)** | **(Yes/No)** | **(Yes/No)** |
| T. Iveson,2019 | Chemotherapy | 65 | 60.5 | 3044 | Ⅱ/Ⅲ | Capecitabine+Oxaliplatin+FU | Yes | Yes | No |
|  | Chemotherapy | 65 | 60.6 | 3044 |  | Capecitabine+Oxaliplatin+FU |  |  |  |
| T. Hata,2021 | Chemotherapy | 68 | 53 | 185 | Ⅱ/Ⅲ | UFT+LV | Yes | Yes | No |
|  | Chemotherapy | 67 | 56.7 | 188 |  | UFT+LV |  |  |  |
| A.     Isogai,2007 | Chemotherapy | NA | 55.9 | 34 | Ⅱ/Ⅲ | UFT | Yes | Yes | No |
| SAKK,1995 | Surgery alone | 62 | 53.4 | 266 | Ⅱ/Ⅲ |  | Yes | Yes | No |
|  | Chemotherapy | 61 | 56.3 | 267 |  | Mitomycin+FU |  |  |  |
| Y. Kasai,1995 | Chemotherapy | 59 | 48 | 327 | Ⅱ/Ⅲ | 5-FU | Yes | Yes | No |
|  | Surgery alone | 61 | 52.3 | 279 |  |  |  |  |  |
|  | Chemotherapy | 58 | 62.6 | 297 |  | 5-FU |  |  |  |
|  | Surgery alone | 60 | 60.1 | 293 |  |  |  |  |  |
| C. Kosugi,2021 | Chemotherapy | 66 | 69.7 | 80 | Ⅱ/Ⅲ | UFT+LV | Yes | Yes | No |
|  | Chemotherapy | 65.5 | 65.8 | 79 |  | UFT+LV+Oxaliplatin |  |  |  |
| T. Yamamoto,1998 | Surgery alone | 60 | 64.9 | 37 | Ⅱ/Ⅲ |  | Yes | Yes | No |
|  | Chemotherapy | 60 | 60.5 | 38 |  | 5-FU+LV |  |  |  |
| L. M. Rousselot, 1968 | Surgery alone | NA | 48.1 | 250 | Ⅱ/Ⅲ |  | Yes | Yes | No |
|  | Chemotherapy | NA | 53.6 | 81 |  | 5-FU |  |  |  |
| J. Li, 2019 | Chemotherapy | 61 | 66 | 87 | Ⅰ/Ⅱ/Ⅲ/Ⅳ | XELOX | Yes | Yes | No |
|  | Chemotherapy | 60 | 62 | 85 | Ⅰ/Ⅱ/Ⅲ | XELOX |  |  |  |
| M.Wunder,1985 | Surgery alone | 64.9 | 50 | 62 | Ⅱ/Ⅲ |  | Yes | Yes | No |
|  | Chemotherapy | 60.1 | 52.5 | 59 |  | 5-FU+MMC+ARA-C |  |  |  |
| G. Portier, 2006 | Surgery alone | NA | 62.4 | 85 | Ⅰ/Ⅱ/Ⅲ/Ⅳ |  | Yes | Yes | No |
|  | Chemotherapy | NA | 53.5 | 86 |  | LV+FU |  |  |  |
| R. Porschen, 2001 | Chemotherapy | 62.6 | 46.2 | 331 | Ⅱ/Ⅲ | Levamisole+5-FU | Yes | Yes | No |
|  | Chemotherapy | 62.4 | 42.7 | 349 |  | 5-FU+LV |  |  |  |
| S. Okabe, 2004 | Chemotherapy | 54 | 65 | 23 | Ⅱ/Ⅲ | CDDP + 5FU + LV | Yes | Yes | No |
|  | Chemotherapy | 61 | 54 | 28 |  | 5FU + LV |  |  |  |
| B. Nordlinger, 2005 | Chemotherapy | 64 | 52 | 471 | Ⅰ/Ⅱ/Ⅲ | Levamisole+FU | Yes | Yes | No |
|  | Chemotherapy | 64 | 58 | 455 |  | FU + LV |  |  |  |
|  | Chemotherapy | 63 | 49 | 465 |  | Levamisole+FU |  |  |  |
|  | Chemotherapy | 63 | 53 | 466 |  | FU + LV |  |  |  |
| G. M. Mavligit, 1976 | Surgery alone | NA | NA | 73 | Ⅲ |  | Yes | Yes | No |
| M. Lorenz, 1998 | Surgery alone | 61 | 64 | 111 | Ⅱ/Ⅲ |  | Yes | Yes | No |
|  | Chemotherapy | 61 | 50.9 | 108 |  | 5-FU+LV |  |  |  |
| W. Lawrence,1975 | Surgery alone | 63.4 | 47 | 76 | Ⅰ/Ⅱ/Ⅲ |  | Yes | Yes | No |
|  | Chemotherapy | 60.7 | 50 | 80 |  | 5-FU |  |  |  |
| W. Lawrence,1978 | Surgery alone | NA | NA | 101 | Ⅰ/Ⅱ/Ⅲ |  | Yes | Yes | No |
|  | Chemotherapy | NA | NA | 102 |  | FU |  |  |  |
| J. Kulig, 2007 | Surgery alone | 58.9 | 70.5 | 102 | Ⅱ/Ⅲ |  | Yes | Yes | No |
|  | Chemotherapy | 58.5 | 64.7 | 102 |  | 5-FU+LV+Irinotecan |  |  |  |
| M. M. Kemeny, 2002 | Surgery alone | 62 | 62.6 | 56 | Ⅱ/Ⅲ |  | Yes | Yes | No |
|  | Chemotherapy | 59 | 73.3 | 53 |  | FUDR+5-FU |  |  |  |
| T. Irvin, 1986 | Surgery alone | 66.6 | 49.3 | 67 | Ⅰ/Ⅱ/Ⅲ |  | Yes | Yes | No |
|  | Chemotherapy | 67.5 | 51.5 | 68 |  | 5-FU |  |  |  |
| T. Hotta,2006 | Surgery alone | NA | 56.3 | 16 | Ⅱ/Ⅲ |  | Yes | No | No |
|  | Chemotherapy | NA | 61.1 | 36 |  | l-LV+5-FU |  |  |  |
| P. Rougier,1998 | Surgery alone | NA | 50 | 599 | Ⅰ/Ⅱ/Ⅲ |  | Yes | Yes | No |
| T. Kato, 2002 | Surgery alone | 61.4 | 53.5 | 144 | Ⅱ/Ⅲ |  | Yes | Yes | No |
|  | Chemotherapy | 60.2 | 53.1 | 145 |  | UFT |  |  |  |
| Q. C. Group,2007 | Surgery alone | 63 | 60 | 1617 | Ⅰ/Ⅱ/Ⅲ |  | Yes | Yes | No |
|  | Chemotherapy | 63 | 62 | 1622 |  | l-LV+FU |  |  |  |
| R. G. Gray, 2000 | Surgery alone | 62 | 58.9 | 2434 | Ⅱ/Ⅲ |  | Yes | Yes | No |
|  | Chemotherapy | 62 | 59.1 | 2464 |  | FU+LV |  |  |  |
|  | Chemotherapy | 62 | 68.1 | 2463 |  | FU+LV |  |  |  |
|  | Chemotherapy | 62 | 59.2 | 2429 |  | FU+levamisole |  |  |  |
| J. Watanabe,2021 | Chemotherapy | 65.5 | 53.3 | 478 | Ⅲ | UFT+LV | Yes | Yes | No |
|  | Chemotherapy | 65 | 55.1 | 477 |  | SOX |  |  |  |
| D. Zhu, 2021 | Chemotherapy | 59 | 56 | 347 | Ⅱ/Ⅲ | FU+Oxaliplatin | Yes | Yes | No |
| T. B. Grage, 1978 | Surgery alone | NA | NA | 113 | Ⅱ/Ⅲ |  | Yes | Yes | No |
|  | Chemotherapy | NA | NA | 98 |  | 5-FU |  |  |  |
| L. P. Fielding, 1992 | Surgery alone | NA | NA | 145 | Ⅰ/Ⅱ/Ⅲ |  | Yes | Yes | No |
|  | Chemotherapy | NA | NA | 123 |  | Heparin |  |  |  |
|  | Chemotherapy | NA | NA | 130 |  | Heparin+5-FU |  |  |  |
| F. Di Costanzo, 2003 | Chemotherapy | 63 | NA | 844 | Ⅱ/Ⅲ | 5-FU | Yes | Yes | No |
|  | Chemotherapy | 63 | NA | 859 |  | LV+5-FU |  |  |  |
| I. Chau,2005 | Chemotherapy | 62 | 54.5 | 404 | Ⅱ/Ⅲ | 5-FU/LV | Yes | Yes | No |
|  | Chemotherapy | 63 | 53.2 | 397 |  | PVI 5-FU |  |  |  |
| Y. Tang, 2020 | Surgery alone | NA | 14.7 | 52 | Ⅰ/Ⅱ/Ⅲ |  | Yes | NA | No |
|  | Surgery alone | NA | 14.2 | 42 |  |  |  |  |  |
| N. Tomita, 2019 | Chemotherapy | NA | NA | 654 | Ⅲ | Capecitabine | Yes | Yes | No |
|  | Chemotherapy | NA | NA | 650 |  | Capecitabine |  |  |  |


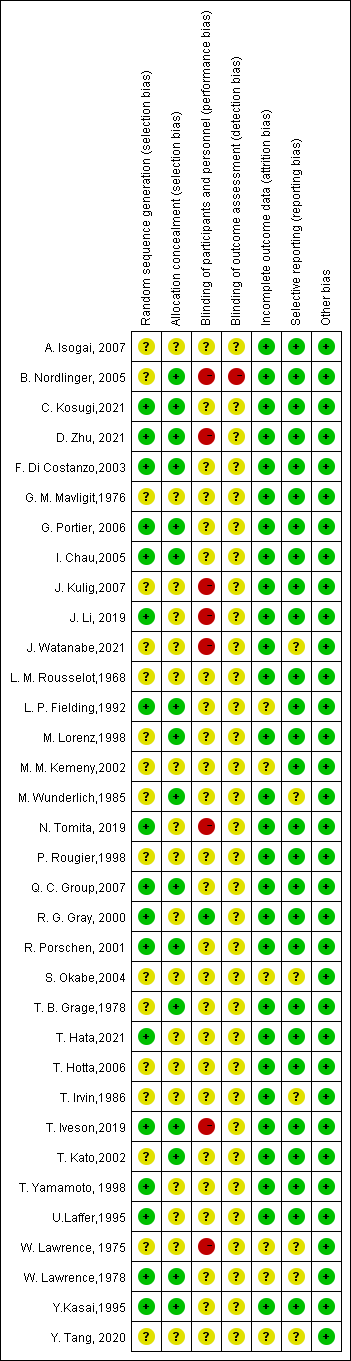


**
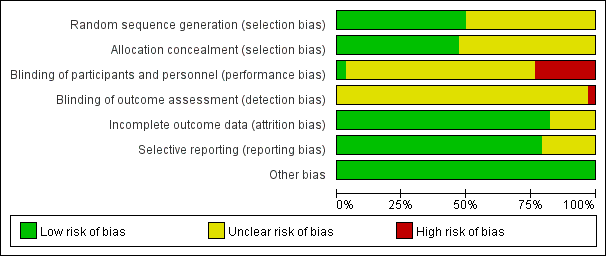
**

**Figure S2.** Risk of bias assessment. Using Cochrane’s risk of bias assessment tool to assess the risk of bias. In this tool, studies were deemed to be at high, low or unclear risk of bias based on adequacy of sequence generation, allocation concealment, blinding, processing of incomplete data, selective reporting, and other biases.

**
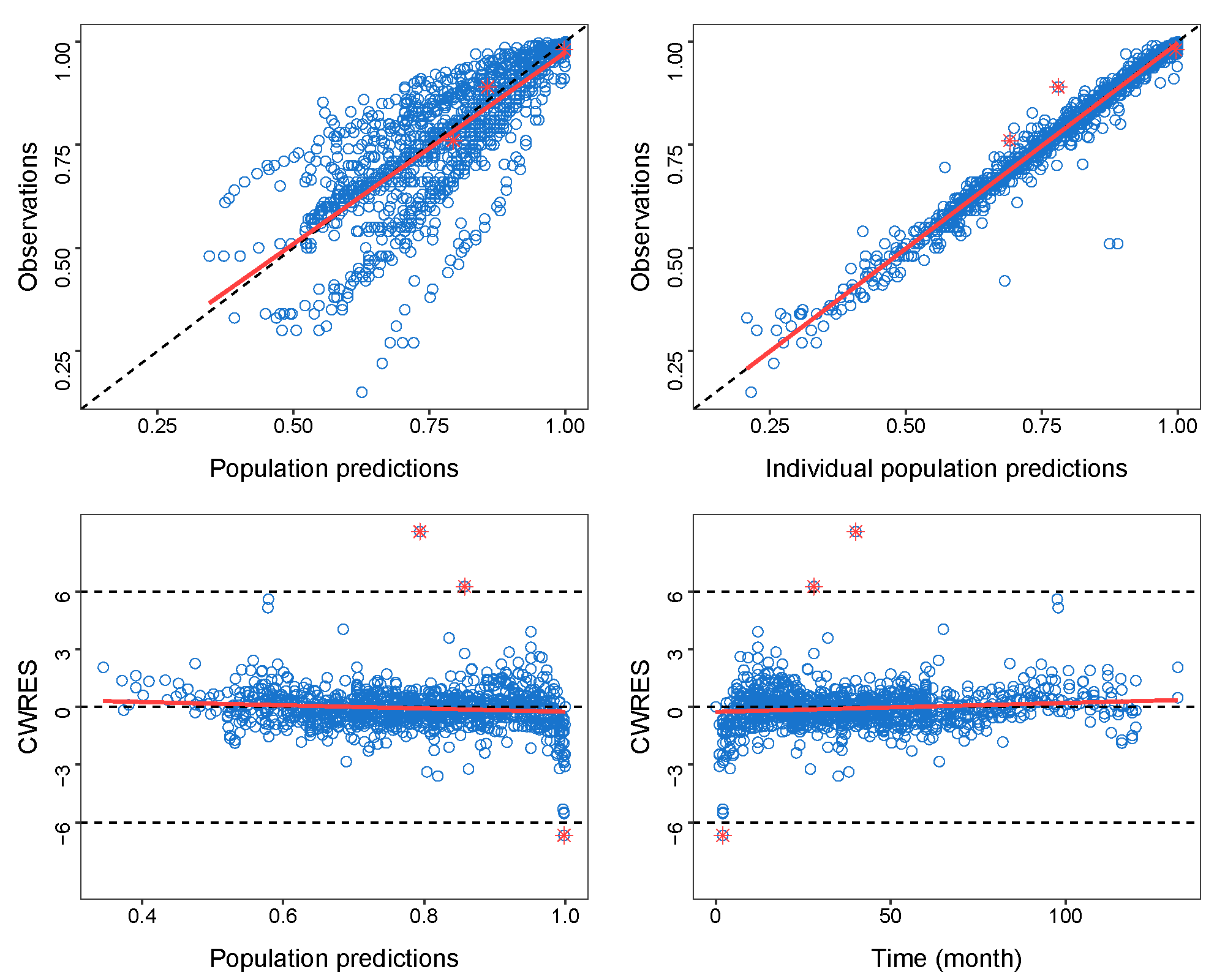
**

**Figure S3.** Goodness of fit plot for OS model. (A) Population predicted value versus observed value. (B) Individual predicted value versus observed value. (C) Conditional weighted residuals versus population predicted value. (D) Conditional weighted residuals versus time. The dashed and solid lines in (A) and (B) represent identity and regression lines, respectively, whereas in (C) and (D), the black lines are the position where conditional weighted residual equal 0 and the red lines are the regression lines.

**
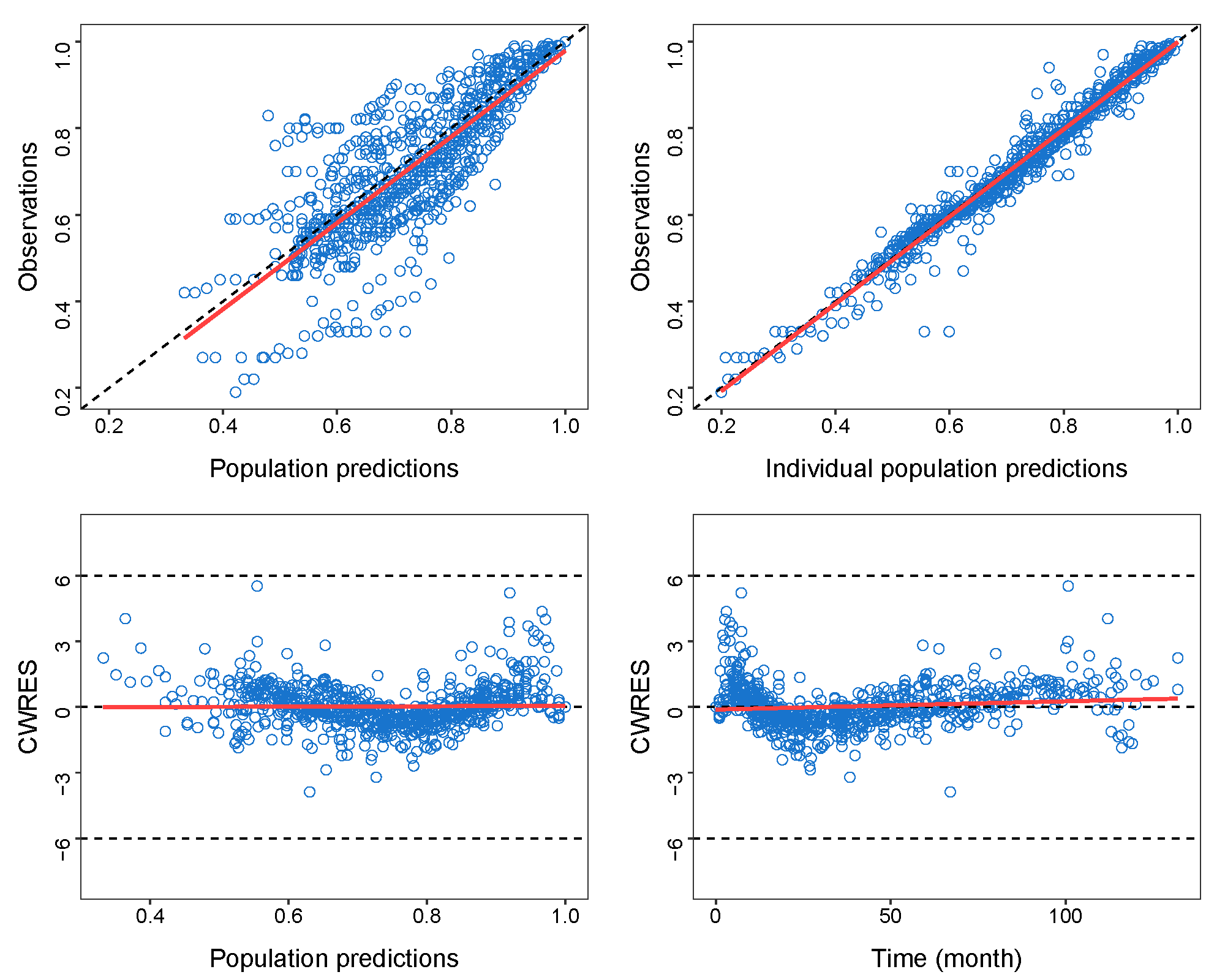
**

**Figure S4.** Goodness of fit plot for DFS model. (A) Population predicted value versus observed value. (B) Individual predicted value versus observed value. (C) Conditional weighted residuals versus population predicted value. (D) Conditional weighted residuals versus time. The dashed and solid lines in (A) and (B) represent identity and regression lines, respectively, whereas in (C) and (D), the black lines are the position where conditional weighted residual equal 0 and the red lines are the regression lines.

**Figure S5. Safety analysis**


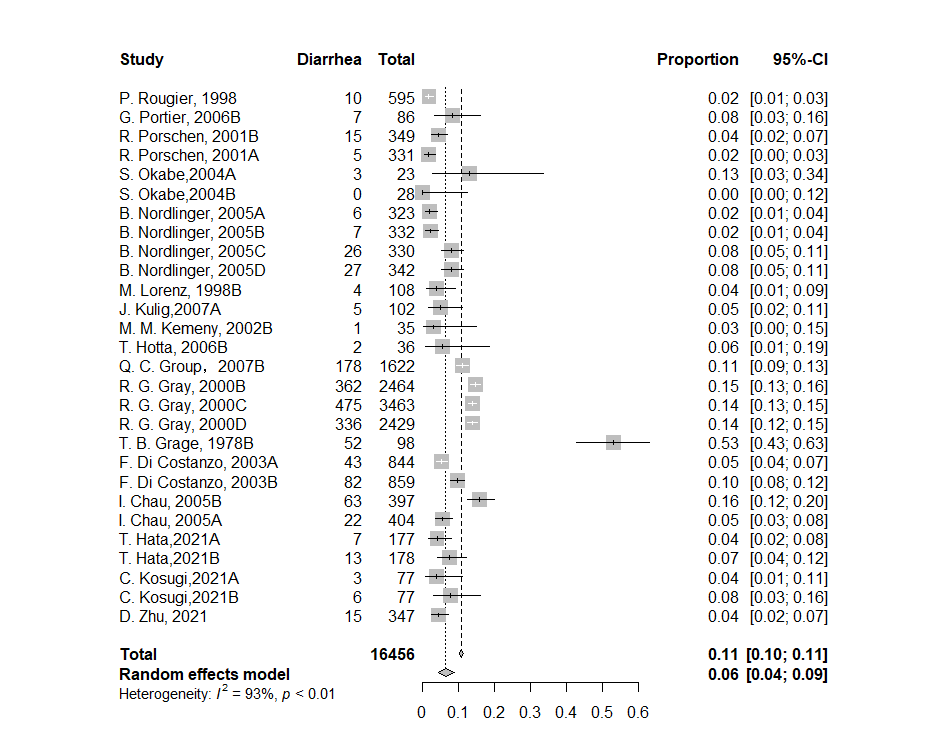

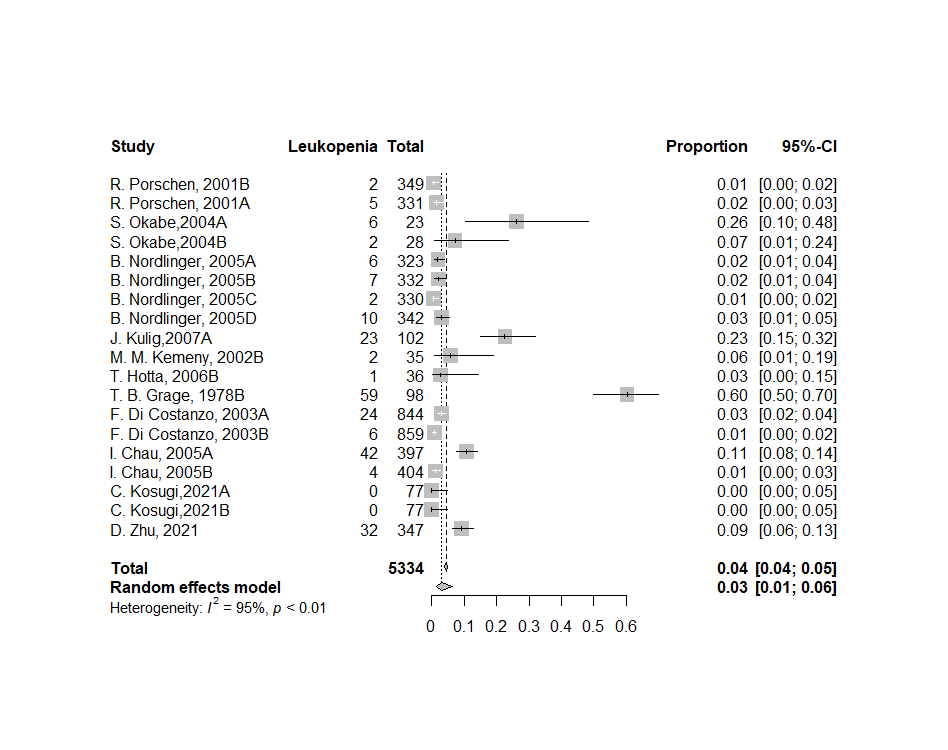

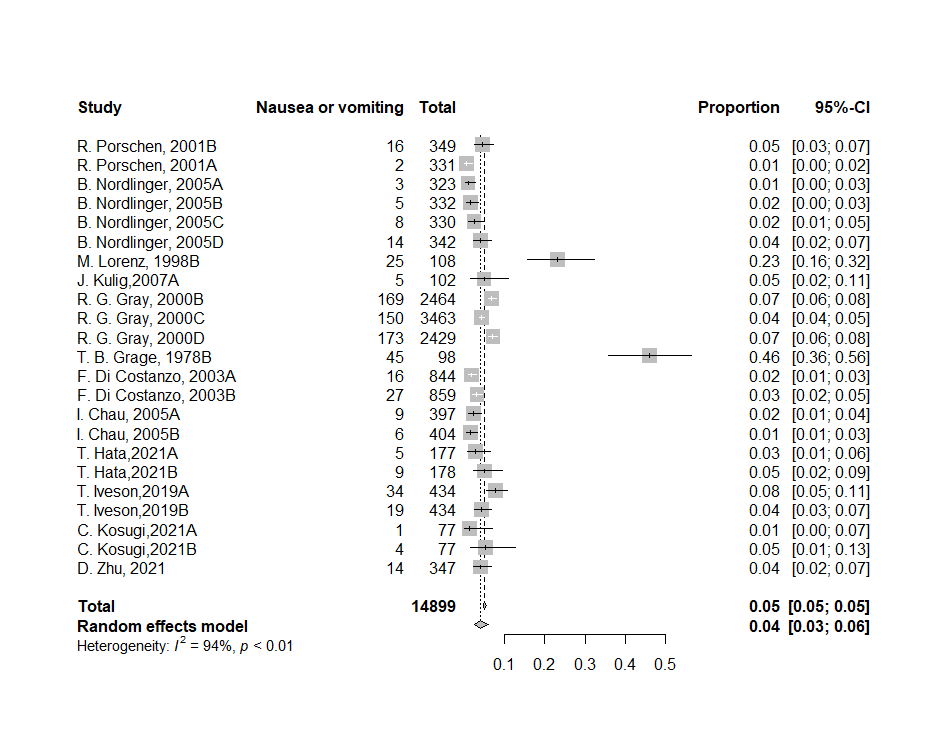

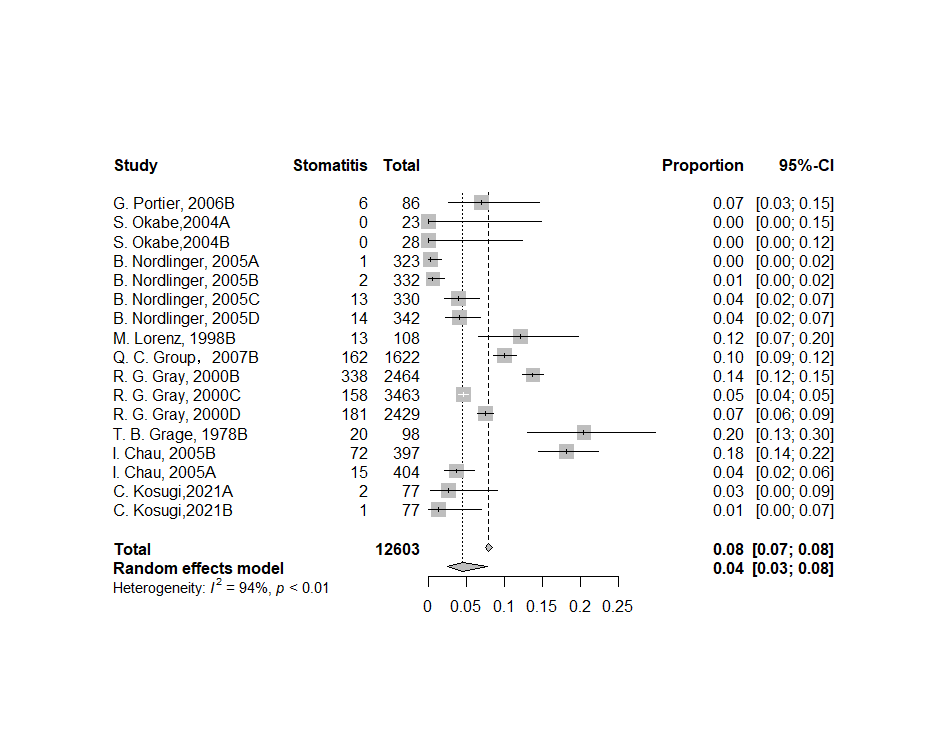

Supplement: Supplementary file 1 [file DataSheet1.docx]
